# Secondary Malignant Neoplasms Following Haematopoietic Stem Cell Transplantation in Childhood

**DOI:** 10.3390/children2020146

**Published:** 2015-04-21

**Authors:** Simon Bomken, Roderick Skinner

**Affiliations:** 1 Department of Paediatric and Adolescent Haematology/Oncology, Great North Children’s Hospital, Newcastle upon Tyne, NE1 4LP, UK; E-Mail: s.n.bomken@newcastle.ac.uk; 2 Children’s Haemopoietic Stem Cell Transplant Unit, Great North Children’s Hospital, Newcastle upon Tyne, NE1 4LP, UK

**Keywords:** children, secondary malignant neoplasm, haematopoietic stem cell transplant, therapy-related acute myeloid leukaemia, post-transplant lymphoproliferative disorders

## Abstract

Improving survival rates in children with malignancy have been achieved at the cost of a high frequency of late adverse effects of treatment, especially in intensively treated patients such as those undergoing haematopoietic stem cell transplantation (HSCT), many of whom suffer the high burden of chronic toxicity. Secondary malignant neoplasms (SMNs) are one of the most devastating late effects, cause much morbidity and are the most frequent cause of late (yet still premature) treatment-related mortality. They occur in up to 7% of HSCT recipients by 20 years post-HSCT, and with no evidence yet of a plateau in incidence with longer follow-up. This review describes the epidemiology, pathogenesis, clinical features and risk factors of the three main categories of post-HSCT SMNs. A wide range of solid SMNs has been described, usually occurring 10 years or more post-HSCT, related most often to previous or conditioning radiotherapy. Therapy-related acute myeloid leukaemia/myelodysplasia occurs earlier, typically three to seven years post-HSCT, mainly in recipients of autologous transplant and is related to previous alkylating agent or topoisomerase II inhibitor chemotherapy. Post-transplant lymphoproliferative disorders occur early (usually within two years) post-HSCT, usually presenting as Epstein-Barr virus-related B cell non-Hodgkin lymphoma.

## 1. Introduction

The increasing long-term survival rates of children with malignancy, reaching 75%–80% in resource-rich countries [1], mean that nearly one in 700 young adults is a survivor of a childhood malignancy [2]. However, such impressive survival rates have been accompanied by significant risks of late treatment-related toxicity. Overall, 60%–75% of long-term survivors have at least one, and 30% at least two, chronic medical problems whilst 30%–40% have a severe, life-threatening or disabling adverse effect [3,4,5,6]. Some groups of survivors, especially those who have undergone more intensive treatment with higher doses of chemotherapy or radiotherapy, have even bigger risks of late adverse effects (LAEs). Survivors of haematopoietic stem cell transplant (HSCT) represent a particularly high risk group. For example, a single-centre cohort study reported an overall cumulative incidence of LAEs of 93% in 162 individuals surviving at least two years after paediatric HSCT, with 32% suffering from four to six late effects and 24% from a severe burden of LAEs, defined as at least seven late effects or at least one severe (CTCAE grade 3) toxicity [7]. The BMT Survivor Study, a US two centre cohort study including both paediatric and adult HSCT survivors, reported cumulative incidences of 71% for any chronic health condition and 41% for severe or life-threatening conditions in 324 10-year survivors [8]. Many treatment-induced late toxicities may have long latent periods between the causative treatment and the development of the toxicity. For example, the cumulative incidence and the risk of mortality attributable to some life-threatening LAEs such as cardiomyopathy and secondary malignant neoplasms (SMNs) continues to rise for several decades after completion of treatment, with no evidence so far of a plateau [9,10].

Although the nature, severity and outcomes of such LAEs vary widely, some have far-reaching consequences. SMNs are one of the most devastating and serious late complications in childhood cancer survivors (CCS). They cause considerable morbidity and represent the commonest cause of treatment-related late (yet still premature) mortality in individuals who may have been cured of their primary malignancy. It is vitally important to understand the nature, risk factors for and causes of LAEs in general, but particularly of the more severe ones such as SMNs, in order to develop better strategies that will reduce the burden of excess morbidity and mortality faced by CCS without compromising the cytotoxic efficacy and success rates of contemporary treatment. Several approaches may be used including primary prevention (e.g., health behaviour changes to partially abrogate the increased risk of SMNs or vascular disease), surveillance (screening) to permit earlier detection of emerging toxicities at a stage when earlier treatment may improve the outcome [11], as well as effective treatment of established LAEs (see Armenian 2012 for a description of these approaches in anthracycline-induced cardiomyopathy [12]).

An important proportion of children with higher risk malignancies undergo a HSCT as part of their first-line treatment. The proportion differs considerably between specific malignancies and has varied greatly across different treatment epochs, depending in part on overall treatment strategies and the perceived benefits and potential risks of transplantation. HSCT in first remission is indicated in patients with acute myeloid leukaemia (AML) or acute lymphoblastic leukaemia (ALL) who have high risk cytogenetic features or high levels of minimal residual disease after induction and/or consolidation treatment. Indications in solid tumours include metastatic or poorly responding Ewing sarcoma and children presenting with high risk (metastatic or MYCN amplified) neuroblastoma. This final indication is the commonest within a specific paediatric malignancy, representing approximately half of all children with neuroblastoma [13]. These children have superior survival when intensive, multi-modality therapy is consolidated with high dose busulphan/melphalan chemotherapy and autologous stem cell rescue [14]. Additionally, HSCT has an important role in the management of relapsed/refractory disease, often in heavily pre-treated children, notably those with leukaemia or lymphoma. An increasing number of patients from both of these groups become long-term survivors and are at very high risk of LAEs for a variety of reasons. These reasons include preceding chemotherapy and/or radiotherapy, used either as conditioning for the transplant or during their earlier cytotoxic treatment, potentially toxic supportive care drugs, and the consequences of other serious complications after HSCT, especially infection and chronic graft-*versus*-host disease (GvHD). These constitute a powerful mix of additive and potentially synergistic chronic toxicities, leading to multiple and often severe late effects, most notably SMNs.

In most cases, SMNs are regarded as occurring secondary to previous treatment, although in some cases other genetic and environmental factors may play important roles in their pathogenesis. Indeed, some children undergoing HSCT for non-malignant conditions (e.g., bone marrow failure) may develop a *first* malignancy that is *secondary* to their previous treatment.

Post-HSCT SMNs fall into three broad categories with important aetiological and clinical characteristics (Table 1) [15]. Depending on their underlying disease and their previous treatment, some groups of patients may be at particularly high risk of one or more of these categories. Two of these categories are clearly related to previous cytotoxic treatment. Secondary, or therapy-related acute myeloid leukaemia/myelodysplastic syndrome (t-AML/MDS) tends to occur relatively early (up to 10, but classically three to five years) after treatment with chemotherapy (typically alkylating agents or topoisomerase II inhibitors), whilst a variety of solid tumours may arise later (usually 10–15 years or more) after treatment with radiotherapy. The third category comprises post-transplant lymphoproliferative disorders (PTLDs), usually manifesting as an EBV-related B cell non-Hodgkin lymphoma (NHL) occurring early post-HSCT (usually within two years).

In addition, a small number of patients undergo HSCT for life-threatening complications of conditions that also confer cancer predisposition. The prototype example is Fanconi anaemia (FA) which usually results in bone marrow failure (aplastic anaemia) necessitating HSCT and which is also associated with a very high risk of premature malignancy, especially squamous cell carcinoma of the head, neck and anogenital mucosal surfaces, in early or middle adulthood [16]. This risk may be aggravated further by HSCT or its complications, especially GvHD [17].

**Table 1:** Features of secondary malignancies following HSCT performed in childhood [13]. Please confirm the highlights.

Characteristics of SMNs	Related to Previous and/or Conditioning Cytotoxic Treatment		PTLD
	Solid Tumours	t-AML/MDS	
Clinical presentation	Wide variety of sites including skin, thyroid, brain, gastrointestinal *etc.*	Cytopenias and associated clinical features of bone marrow failure, or overt AML with fever, lymphadenopathy, coagulopathy *etc.*	Spectrum from Glandular fever-like illness, lymphadenopathy, lymphomatous deposits, fever and organ dysfunction. Rarely primary CNS lymphoma
Incidence	7% at 20 years post-HSCT, but still rising by further 2% for every subsequent 5 years additional follow-up	1%–2% following autologous transplant, but higher with intensive pre-transplant chemotherapies. Rarely described post allogeneic HSCT	Approximately 1% overall, up to 70% with multiple risk factors
Latency	Variable but often long—Up to at least 20–30 years	Variable from 6 months for t-AML secondary to topoisomerase II inhibitors to 8–10 years for alkylating agent induced t-MDS	Short—Most cases present in first year post transplant
Age at presentation	Any	Any	Any
Associated features	Continued rise in cumulative incidence even after >20 years post-HSCT	Topoisomerase II inhibitors associated with an increased incidence of rearrangements of, classically, 11q32, but also rearrangement of 21q22, t(3;21)(q26.2;q22.1), t(15;17)(q22;q12) or inv(16)(p13.1q22). Alkylating agents associated with an increased incidence of unbalanced cytogenetic changes, especially del(7q) and del(5q)	EBV viraemia, fever, organ dysfunction
Risk factors	Radiotherapy during previous treatment or conditioning regimen GvHD (especially chronic)	Epipodophyllotoxin/alkylating agent use PBSC (*versus* marrow) as stem cell source?	Specific T cell depletion > non-specific lymphocyte depletion. Intense immunosuppression (after RIC HSCT or for treatment of GvHD). Unrelated or mismatched related donor

HSCT—Hematopoietic stem cell transplantation; GvHD—Graft *versus* host disease; t-therapy related; AML—acute myeloid leukaemia; MDS—Myelodysplasia; PBSC—Peripheral blood stem cell; PTLD—Post transplant lymphoproliferative disease; CNS—Central nervous system; EBV—Epstein-Barr virus; RIC—Reduced intensity conditioning.

## 2. Epidemiology

### 2.1. Secondary Malignant Neoplasms in Childhood Cancer Survivors

Nearly all the early reports of SMNs in the late 1970s and 1980s were in CCS (rather than survivors of adult malignancy), at least in part due to the higher survival rates in this age group which allowed more opportunity to develop LAEs in general, including SMNs [18,19]. Although it is now recognised that SMNs occur in all age groups, realisation of their true frequency has only emerged in the last 15–20 years.

Although the *relative risk* (RR) of developing a SMN (*i.e.*, the increased risk of developing a malignancy expressed as a ratio to the risk in a healthy age-matched population) in CCS is relatively high, the *absolute risk* remains low since malignancy is rare *per se* in childhood. Conversely, even though the RR is higher in children than in adults, the absolute frequency of SMNs is higher in the adult population because primary malignancies are much commoner in adulthood. Other statistical measures employed to quantify SMNs have included the standardised mortality ratio (SMR—an age and gender-standardised measure of mortality expressed as a ratio to the mortality rate of the general population) and standardised incidence ratio (SIR—a similarly standardised measure of incidence expressed as a ratio to the incidence rate in the general population).

The first large studies in CCS suggested that about 1% of five-year survivors developed a SMN [18,19]. Subsequent longer-term single-centre and larger population-based cohort studies from Europe and the USA, with post-treatment follow-up extending up to 30 years, reported SMNs in 4%–13% of survivors, representing RRs of approximately 4–15 [19,20,21,22,23,24]. Further studies have revealed the implications of these SMNs on long-term mortality. Very long-term data from over 20,000 five-year survivors in the North American multi-institutional Childhood Cancer Survivor Study (CCSS) cohort, followed up for 16–32 years from initial diagnosis, revealed that they had a SMR of 8.4. Although 57.5% of deaths occurring more than five years from initial diagnosis were due to recurrence of the primary malignancy itself, most of the remainder resulted from LAEs of treatment, with 18.6% being due to second or subsequent malignant neoplasms (SMR 15.2), followed by cardiac (6.9%) and pulmonary (2.6%) complications [25]. Moreover, 25 years after diagnosis of childhood malignancy, the death rate due to a subsequent malignancy exceeded that due to all other causes combined. This finding was confirmed by a separate study of nearly 18,000 five-year survivors in the population-based British Childhood Cancer Survivor Study (BCCSS) cohort, in which SMNs accounted for over 50% of the *excess risk* of mortality beyond 45 years from original diagnosis [9].

### 2.2. Secondary Malignant Neoplasms in Survivors of HSCT

Over the last two decades, several large studies have demonstrated a particularly high risk of SMNs in survivors of both adult and childhood HSCT, reporting incidences of up to 10%–15% by 15 years post-transplant and highlighting concern that the increased risk of solid SMNs does not plateau even after this length of follow-up. Several different patient populations have been studied retrospectively, ranging from single institution to multicentre registry cohorts, all HSCT survivors or specifically those of allogeneic or autologous transplants or of HSCTs performed for specific indications (e.g., acute leukaemia, neuroblastoma), or in specific age groups (e.g., children or adult). Not only have these studies addressed different issues relevant to the specific patient and transplant populations, but the contrasting characteristics of these populations have inevitably led to diverse findings regarding the epidemiological features of, and risk factors for, post-HSCT secondary malignancies. Table 2 summarises the characteristics and major results of studies that have described and analysed SMNs occurring after HSCT performed in childhood.

Several large studies, particularly in survivors of adult HSCT, have described the increased risks of solid SMNs in general after HSCT [26,27,28,29,30,31,32,33,34,35,36], whilst some have particularly highlighted the risks of oral [37], skin (including melanoma, squamous cell carcinoma [SCC] and basal cell carcinoma [BCC]) [28,33,38], gastrointestinal [39] and thyroid [40,41] SMNs. A number of studies have highlighted the risk of haematological SMNs especially t-AML/MDS [27,28,29,30,42], predominantly in survivors of autologous HSCT. PTLD, whilst not unique to HSCT survivors, has been well characterised after allogeneic HSCTs [27,35,42,43].

Several large studies have demonstrated a cumulative risk of solid SMNs of 10%–15% at 15 years post-HSCT using Kaplan-Meier analysis, although more recent studies using competing risks analysis have shown a lower magnitude of risk of 3%–7%. Early large registry studies used Kaplan-Meier analysis to provide important information about the types of SMNs experienced by HSCT survivors. A study of five year survivors of autologous or syngeneic (*n* = 79) or allogeneic (*n* = 903) HSCT survivors transplanted consecutively in 45 European centres before 1986, approximately half of whom were transplanted at ≤20 years of age, reported actuarial incidences of all SMNs of 3.5% at 10 years and 12.8% at 15 years, with a RR of 3.8 [32]. A study of 3,182 survivors of allogeneic HSCT for childhood acute leukaemia performed in 235 centres between 1964 and 1992 also found a high and increasing cumulative incidence of SMNs reaching 11% at 15 years, with brain and thyroid tumours accounting for 56% of these [35]. In contrast, a more recent very large multicentre international registry study of 28,874 patients undergoing allogeneic HSCT between 1964–1996 for leukaemia and other severe haematological diseases used competing risks analysis and found a cumulative incidence of solid SMNs (excluding PTLD, BCCs and haematological malignancy) of 3.3% at 20 years post-HSCT [33].

Large single centre studies have provided additional data. One study of 2,129 survivors of HSCT (64% allogeneic) performed predominantly for haematological malignancies used Kaplan-Meier analysis to demonstrate that the incidence of solid SMNs continued to rise to 15% at 15 years post-HSCT, with the expectation of additional increases with further follow-up [26]. In contrast, an update of the large Minneapolis single centre cohort originally described by Bhatia [27] used competing risks analysis and found a cumulative incidence of SMNs of 6.9% at 20 years post-HSCT, most of which were solid tumours [42]. However, the incidence continued to rise by about 2% for every five years of further follow-up.

An analysis of 605 adult patients undergoing autologous bone marrow transplantation for NHL after conditioning treatment with cyclophosphamide and total body irradiation (TBI) demonstrated significant risks both of t-AML/MDS and of solid SMNs. Using competing risks analysis, the overall incidence of SMNs (including t-AML/MDS) was 21% by 10 years, split approximately equally between t-AML/MDS and other SMNs (including solid tumours and non-MDS haematological malignancies) [29]. Again, the incidence of solid tumours continued to rise with longer follow-up leading to a projected incidence of all SMNs of 29% by 15 years.

**Table 2:** Secondary malignancies following haematopoietic stem cell transplantation (HSCT) performed in childhood *.

Author, Date	Study Characteristics	Secondary Malignancies	Risk Factors	Comments
**Paediatric Studies**				
Socie, 2000 [35]	Multicentre international registry (IBMTR) and Seattle. Allogeneic HSCT for acute leukaemia. 3182 patients, HSCTs performed 1964–1992. Kaplan-Meier analysis of probability of SMN. Uni- and multivariate analysis of risk factors	45 invasive SMNs (25 solid tumours, 20 PTLDs) compared with 1 expected case (*p* < 0.001). Solid tumours included brain (9), thyroid (5), melanoma (3), tongue SCC (3), salivary gland carcinoma (2), osteosarcoma (2)	Solid tumours associated with age <10 years at HSCT (RR 3.7) (especially for brain and thyroid SMNs, RR 12.2) and high dose TBI (>10 Gy single fraction, >13 Gy fractionated) (RR 3.1). NB Chronic GvHD or cGvHD lowered risk of solid tumours (RR 0.2). PTLD associated with moderate or severe cGvHD (RR 6.5), unrelated or HLA mismatched related donor (RR 7.5), T cell depletion (RR4.8) and use of ATG (RR 3.1)	Cumulative incidence of invasive solid SMN 0.9%, 4.3% and 11% at 5, 10 and 15 years post-HSCT respectively. Latency—median 6.0 (range 0.3–14.3) years
Danner-Koptik, 2013 [30]	Multicentre international registry (CIBMTR). Autologous HSCT for lymphomas or solid tumours. 1487 patients, HSCTs performed 1987–2003. Competing risks (cumulative incidence function) analysis of probability of SMN. Multivariate analysis of risk factors	35 SMNs (13 AML/MDS, 20 solid tumours). Solid tumours included bone (5), thyroid (5), breast (2), soft tissue (2). SIRs—overall 24, AML 266, MDS 6,603, bone 81, thyroid 53, breast 93, soft tissue 34	No association between risk of SMN and age, gender, diagnosis, remission status at HSCT, time from diagnosis to HSCT, TBI, etoposide in conditioning, any radiotherapy, number of HSCTs, year of HSCT	Cumulative incidence of SMNs 1.0% and 2.6% at 5 and 10 years post-HSCT (0.6% and 1.1% AML/MDS, 0.4% and 1.3% solid tumours). Latency—6.3 (0.4–20.4) years (AML/MDS—2 years, solid tumours—7 years)
De Latour, 2014 [44]	Multicentre international registry (EBMT). First HLA-matched allogeneic HSCT for Fanconi anaemia. 796 patients, HSCTs performed 1972–2009 (49% since 1999). Competing risks (cumulative incidence function) analysis of probability of SMN. Multivariate analysis of risk factors	30 SMNs (only 2 since 2000). 89% were solid tumours, including 20 SCCs (13 were oral/oesophageal)	10–20 or >20 years age at HSCT (HR 2.3 and 3.3 respectively). Clonal evolution as indication for HSCT (HR 4.6). PBSC as stem cell source (HR 3.3). Previous cGvHD (HR 3.3). Radiotherapy and donor type were not risk factors	Cumulative risk of SMNs 21% and 34% at 15 and 20 years post-HSCT respectively (in the 509 patients who survived >1 year post-HSCT)
Martin 2014 [45]	Single centre retrospective analysis of 87 children undergoing autologous HSCT for high risk neuroblastoma. Competing risks analysis of probability of SMN. Univariate analysis of risk factors	10 SMNs, including t-AML (1), t-MDS (5), papillary thyroid carcinomas (2), chondrosarcoma followed by hepatocellular carcinoma (1), biliary adenocarcinoma (1)	High risk of t-AML/MDS in this study likely to reflect the very high dose of cyclophosphamide and etoposide compounded by late harvesting of PBSCs	Cumulative incidence of SMN 7.2%, 16.5% and 34.2% at 5, 10 and 15 years respectively
Bhatia, 1996 [27]	Single centre USA. Autologous and allogeneic HSCT for any diagnosis. 2150 patients, aged 20.0 (0.2–67) years at HSCT performed 1974–1995. Kaplan-Meier analysis of probability of SMN. Uni- and multivariate analysis of risk factors	53 SMNs in 51 patients (22 PTLDs, 17 solid tumours, 11 AML/MDS, 3 lymphoma). Solid tumours included melanoma (3), BCC (3), brain (2). SIRs—overall 11.6, solid tumours 3.2, melanoma 10.3, brain 11.8, AML/MDS 286	Solid tumours—TBI (RR 6.0) (cGvHD not risk factor for skin SMNs). AML/MDS—PBSC as stem cell source (RR 5.8), age >35 years at HSCT (RR 3.5). PTLD—*in vitro* T cell depletion (RR 11.9), ATG in conditioning (RR 5.9), mismatched donor (RR 8.9), PID (RR 2.5)	Cumulative risk of SMNs 9.9% at 13 years post-HSCT. Solid tumours 5.6% at 13 years. AML/MDS plateaued at 2.1% at 9 years, PTLD at 1.6% at 4 years. Latency—solid tumours 4.0 (0.2–13), AML/MDS 3.0 (0.3–9) years, PTLD 0.2 (0.1–3)
**Mixed Paediatric/Adult Studies**				
Kolb, 1999 [32]	Multicentre international registry (EBMT). Autologous and allogeneic HSCT for leukaemia, lymphoma, inborn errors or aplastic anaemia. 1,036 patients surviving >5 years post-HSCT, aged 21 (1–51.9) years at HSCT performed before 1985. Kaplan-Meier analysis of probability of SMN. Uni- and multivariate analysis of risk factors	53 SMNs including skin (14), oral (7), gastrointestinal (5), thyroid (5), uterine/cervix (5), breast (4), brain (3), leukaemia (1)	Age at HSCT. Immunosuppressive treatment for cGvHD, specifically Cyclosporin (RR 2.5) Thalidomide (RR 3.4)	Cumulative incidence of SMNs 3.5% and 11.5% at 10 and 15 years post-HSCT respectively
Curtis 1999 [46]	Multicentre international registry (CIBMTR) and Seattle. Allogeneic HSCT, excluding patients with primary immune deficiency, inherited cancer predisposition syndromes, initial diagnosis of NHL. 18,014 patients, aged 25 (<1–72) years at HSCT performed 1964–1992. Competing risks analysis for probability of PTLD. Multivariate analysis of risk factors for PTLD	78 cases of PTLDs with 64 (82%) within the first year	Unrelated or HLA-mismatched related donor (RR 4.1). T-cell depletion of donor marrow (RR 12.7). Use of antithymocyte globulin (RR 6.4) or anti-CD3 monoclonal antibody (RR 43.2). Weaker association with grade II-IV aGvHD (RR 1.9) and radiation based conditioning (RR 2.9). Multiplicative effect of risk factors with 2 major risk factors (RR 8.0) and ≥3 factors (RR 22%)	Cumulative incidence of PTLDs 1.0% at 10 years post-HSCT. Latency—Most PTLDs early-onset (64/78 occurred within first year post-HSCT), minority late-onset (14/78 occurred 1–8.6 years post-HSCT)
Bhatia, 2001 [26]	Single centre USA. Autologous and allogeneic HSCT for any diagnosis. 2129 patients, aged 33.9 (1.5–71.5) years at HSCT performed 1976–1998. Kaplan-Meier analysis of probability of SMN. Uni- and multivariate analysis of risk factors. Nested case control study of risk factors	29 solids SMNs. Excluded PTLD and haematological malignancies. Included non-melanoma skin (9) (SCC 3, BCC 6), cervix (4), salivary gland (3), oral SCC (3), breast (2), liver (2), thyroid (2). Overall SIR (excluding skin SCC and BCC) 5.3 if <34 years age at HSCT, only 1.1 if older	Case control study revealed no association between risk of SMN and pre-HSCT or conditioning radiotherapy or chemotherapy, aGvHD or cGvHD, primary diagnosis, age at and type of HSCT However All 6 skin SCC patients had cGvHD Both liver cancer patients had history of chronic hepatitis C infection	Cumulative incidence of SMNs 1.6% and 6.1% at 5 and 10 years post-HSCT respectively (allogeneic 6.4% and autologous 1.6% at 10 years). Latency—No data overall, but cervix 3.3 (1.6–9.7), oral 7.6 (4.7–11.7), breast 9.9 (2.6–17.1), thyroid 12.7 (7.5–18.0)
Baker, 2003 [42] (NB Update of Bhatia 1996) [27]	Single centre USA. Autologous and allogeneic HSCT for any diagnosis. 3372 patients, aged 24 (0.1–67) years at HSCT performed 1974-2001. Competing risks analysis of probability of SMN. Multivariate analysis of risk factors	147 invasive SMNs in 137 patients (44 PTLDs, 62 solid tumours, 36 AML/MDS, 5 other leukaemia/lymphoma. Solid tumours included skin BCC and SCC (19), melanoma (8), carcinoma-*in-situ* (5), oral (5), brain (4), breast (4), lung (3). SIRs—overall 8.1, solid tumours 2.8, AML/MDS 300; if <10 years age at HSCT, overall 60.4, solid tumours 33.3	Solid tumours—Age ≥20 years at HSCT (RR 2.0). TBI not significant^1^. PTLDs—Mismatched related donors (RR 9.0), PID (RR 2.7), CML (RR 2.5), use of ATG (RR 3.7), *in vitro* T cell depletion (RR 4.0), grade 3–4 aGvHD (RR 2.4). AML/MDS—30 of 34 had undergone autologous HSCT. Risk highest for PBSC (RR 3.1)	Cumulative incidence of SMNs 6.9% at 20 years post-HSCT, increasing by ~2% in each successive 5 year follow-up period. Mostly solid tumours after 5 years (3.8% at 20 years). Latency—Very similar to Bhatia, 1996
Cohen, 2007 [40]	Multicentre international registry (EBMT). Autologous and allogeneic HSCT for any diagnosis. 70,859 patients, 27% aged 0–20 years age at HSCT, performed 1985–2003. Competing risks analysis of probability of SMN. Multivariate analysis of risk factors	32 thyroid carcinomas (papillary cell 23, follicular 9). Presented with palpable nodule in 18 (56%). No symptoms/signs and diagnosed only on ultrasound surveillance in 9 (28%). SIRs—Overall 3.3, males 4.1, females 2.9, 0–10 years age at HSCT 61	Age at HSCT (RR 24.6 for <10 years *vs.* >20 years). TBI or thoraco-abdominal radiotherapy (RR 3.4). Females (RR 2.8 *vs.* males). cGvHD (RR 2.9)	Cumulative incidence of thyroid SMNs ~0.05% at 20 years post-HSCT (0.2% in patients 0–10 year age at HSCT). Latency—8.5 (0.6–18.5) years
Landgren 2009 [47]. (NB Update of Curtis 1999) [46]	Multicentre international registry (CIBMTR) and Seattle Allogeneic HSCT, excluding patients with primary immune deficiency, inherited cancer predisposition syndromes, initial diagnosis of NHL. 26,901 patients, aged 26.6 (0.1–68) years at HSCT performed 1964–1996. Competing risks analysis for probability of PTLD. Multivariate analysis of risk factors for PTLD	127 PTLDs identified, 105 (83%) within the first year following transplant.	T cell depletion (RR 3.1–9.4), use of ATG (RR 3.8), HLA mismatching in presence of T-cell depletion/ATG use (RR 3.8), acute (RR1.7) and chronic (RR 2) GvHD. Less effect when T-cell depletion used agents which also remove B cell	Overall observed to expected (O/E) ratio 29.7 (4.2 at ≥5 years post-HSCT). Demonstrated a multiplicative effect of multiple major risk factors on incidence: 0 factors—cumulative risk 0.2% at 12 years post-HSCT; 1 factor—1.1%; 2 factors—3.6%; 3 factors—8.1%. Latency—most PTLDs early-onset (105/127 [83%] occurred within first year post-HSCT), minority late-onset (22/127 occurred 1–>10 years post-HSCT)
Rizzo, 2009 [33]	Multicentre international registry (CIBMTR) and Seattle. Allogeneic HSCT for haematological malignancy, aplastic anaemia (except Fanconi anaemia) and haemoglobinopathy. 28,874 patients, aged 27 (0.08–72.4) years at HSCT performed 1964–1996. Competing risks analysis of probability of SMN (also performed Kaplan-Meier analysis for comparison with previous studies). Multivariate analysis of risk factors	189 SMNs. Excluded BCC, PTLD and haematological malignancies. Included oral/pharyngeal (27), melanoma (18), brain (18), thyroid (16), breast (13), female genital (9), bronchus/lung (8), liver (7), soft tissue (7), bone/joint (6). SIRs—overall 2.1, bone/joint 8.5, oral 7.0, soft tissue 6.5, liver 6.3, brain 5.9, thyroid 5.8, melanoma 3.5	Conditioning radiotherapy (TBI or limited field)—non-SCC tumours (RR 2.3 for TBI). Significant interaction with age at HSCT – RR 55.3 for <10 years, 6.2 if 10–19 years, 4.8 if 20-29 years; no excess risk if >30 years. cGvHD—SCCs (skin RR 11.0, oral RR 5.3). Patient gender (male)—SCCs (skin RR 11.9, oral RR 2.8). Most cases (27/40) of brain/CNS, thyroid, bone, soft-tissue SMNs occurred in patients who underwent HSCT at <17 years age	Cumulative incidence of solid SMNs 3.3% (competing risks analysis) and 8.8% (Kaplan-Meier analysis) at 20 years post-HSCT. Latency—65 SMNs occurred 1–4 years and 100 ≥5 years post-HSCT

* Studies including at least 500 HSCTs, of which ≥10% performed in children. ATG = anti-thymocyte globulin; BCC = basal cell carcinoma; aGvHD/cGvHD = acute/chronic graft-versus-host disease; CIBMTR = Centre for International Blood and Marrow Transplant Research; EBMT = European Society for Blood and Marrow Transplantation; HR = hazard ratio; IBMTR = International Bone Marrow Transplant Registry Database; NHL = non-Hodgkin lymphoma; PBSC = peripheral blood stem cells; PID = primary immunodeficiency; RR = relative risk; SCC = squamous cell carcinoma; SIR = standardised incidence ratio; TBI = total body irradiation. Patient age at HSCT and latency expressed as median (range). ^1^ 78% of patients had TBI or total lymphoid irradiation, which may therefore have reduced power to detect their effect as risk factors.

## 3. Pathogenesis, Clinical Features and Management

### 3.1. Solid Malignancies

A spectrum of solid malignancies is seen following HSCT, with the commonest being skin, oral, uterine, thyroid and breast cancer and glioblastoma [32,33]. Whilst their clinical presentation is not dissimilar to their *de novo* counterparts, there is very little information available concerning the optimal strategies to treat SMNs in survivors of childhood HSCT. The authors of a single centre retrospective analysis suggested that frequent clinical follow-up, based on knowledge of the most likely SMNs, may permit more timely diagnosis and hence earlier stage of malignancy at diagnosis, thereby offering the possibility of improved outcomes [48]. Appropriate and timely surveillance will be an important component of this approach [11]. However, although some studies have demonstrated a reasonably good overall outcome in solid SMNs, the reported survival times are short in those who do die from their secondary malignancy. For example, both Bhatia’s single centre study and Socie’s large multicentre registry study reported that only 40% of patients died from a solid SMN, but stated that the SMN-related deaths occurred at a median of 1.5 months in Bhatia’s study, and at between 0.2–2.7 years in Socie’s [27,35]. Taken together with the many different types of tumours that have been reported, this suggests that there is a wide spectrum in the clinical nature and behaviour of solid SMNs after HSCT, probably reflecting different biological mechanisms.

However, there has been a notable paucity of research into the pathogenetic mechanisms underlying solid SMNs in CCS in general, not just in survivors of HSCT, although radiotherapy is believed to be the major determinant of risk. For example, Rizzo’s recent very large international multicenter registry study was in agreement with previous reports that conditioning radiotherapy, predominantly TBI, is an independent risk factor for solid SMNs post-HSCT [27,35,40], and also confirmed that secondary breast cancer is one of the commoner solid malignancies in this setting [33]. Radiation is a well-documented cause of breast cancer, for example in CCS previously treated with radiotherapy to a field including breast tissue [49] and in atomic bomb survivors [50]. Recent work with secondary breast cancer is beginning to shed more light on molecular genetic mechanisms that underlie radiation-induced malignancy, demonstrating that breast epithelium cell lines and primary cell cultures exposed to ionising radiation display particular sensitivity at the *c-MYC* locus (encoding an oncogenic transcription factor), resulting in mutations, notably high level focal amplification, and increased protein expression. Furthermore, the frequency and magnitude of *c-MYC* amplification and protein expression were significantly higher in tissue from radiation-related breast cancers compared to that in cases unrelated to radiation. The authors concluded that mutation at the *c-MYC* locus is implicated in the pathogenesis of a substantial proportion of radiation-related breast cancer [51].

Chronic GvHD (cGvHD), and hence the necessity for often prolonged immunosuppressive treatment, is also believed to be an important factor in the development of some solid SMNs, particularly mucocutaneous malignancies [32,33]. This appears to be particularly important in patients undergoing HSCT for FA [17].

Although previous alkylating agent treatment may contribute to the development of bone SMNs in CCS in general [52], there is no clear evidence to support the relevance of chemotherapy in the development of post-HSCT SMNs.

As mentioned above, and in stark contrast to the plateau in incidence seen with t-AML/MDS and PTLD, the risk of radiotherapy-associated solid SMNs continues to increase with longer follow-up after HSCT, with latency periods of up to 15 years or even longer [33,42]. Clearly, this has important implications for surveillance strategies. As the likelihood and duration of survival continues to improve after HSCT, it is likely that the full impact of post-HSCT solid SMNs will only be revealed in future decades [53].

### 3.2. Myeloid Malignancies—Acute Myeloid Leukaemia and Myelodysplasia

Therapy-related AML/MDS is predominantly associated with autologous HSCT, occurring mostly within the first 10 years after transplant [27,29]. In this setting, the reconstituting haematopoietic stem cells (HSCs) have frequently been exposed to cytotoxic chemotherapy prior to harvesting and storage. Additionally, many high dose chemotherapy regimens, for example BEAM (carmustine [BCNU] etoposide, cytarabine, melphalan) which is frequently used in relapsed paediatric non-Hodgkin lymphoma patients, are not truly myeloablative. This allows for the possibility of heavily pre-treated and conditioned HSCs undergoing dysplastic and malignant change leading to t-AML/MDS.

Although the occurrence of t-AML/MDS is thought to reflect the cumulative effects of prior chemotherapy/radiotherapy, conditioning and replicative stress, dissecting the respective contributions of these factors has proved challenging. The biggest difficulty is the variation in both the previously administered therapies, many of which are themselves associated with a risk of t-AML/MDS, and the high dose chemotherapy conditioning regimens used for autologous HSCT. Additionally, t-AML/MDS has been reported occasionally following allogeneic HSCT [54,55], a clinical situation where the reconstituting HSCs should not have experienced pre-transplant toxicity. Several explanations exist for this finding—second malignancies may derive from a residual recipient haematopoietic precursor, or the pre-treated microenvironment contributes to the development of t-AML/MDS, or there is an impact of replicative stress on subsequent malignant change. The latter two possibilities are further supported by a relatively high incidence of donor-derived myeloid malignancy post allogeneic HSCT, despite the donor remaining well [27,34,42,43].

The WHO recognises two subsets of therapy-related myeloid malignancies, those resulting from prior use of alkylating agents and those resulting from topoisomerase II inhibitor use [56]. Alkylating agents, such as melphalan, chlorambucil, dacarbazine and cyclophosphamide classically result in a myelodysplastic syndrome with a peak incidence at five to eight years (range 1–20 years) following treatment. Frequently associated with high risk, unbalanced cytogenetic changes, notably del(7q) or del(5q), t-MDS has a higher frequency of conversion to AML and does so over a shorter period than does spontaneous MDS, with a median time of six months.

In contrast, second malignancy associated with topoisomerase II inhibitors (etoposide, teniposide, anthracyclines) classically presents with a shorter latency of two to three years, usually as t-AML without a preceding period of myelodysplasia. This condition is typified by rearrangement of the mixed lineage leukaemia (*MLL*) gene at 11q32, or rearrangement of 21q22, t(3;21)(q26.2;q22.1), t(15;17)(q22;q12) or inv(16)(p13.1q22) [56].

Current management approaches focus on allogeneic HSCT, with or without prior standard chemotherapy, where residual toxicity and suitability of potential donors allows. However, outcome in this group of patients remains poor [27,42]. Bhatia found that eight of 11 patients (73%) with t-AML/MDS died [27], whilst an updated report of this large single centre cohort demonstrated a one-year survival rate of 34% [42].

### 3.3. Post-Transplantation Lymphoproliferative Disorder

PTLD represents a spectrum of disease associated with the period of immune suppression following either haematopoietic stem cell or solid organ transplantation. In keeping with this, the majority of PTLD is associated with viral infection, predominantly Epstein Barr virus (EBV) which is identified in 55%–80% of patients overall, but approaching 100% of those with early onset PTLD, occurring within the first twelve months after transplant [57,58,59]. Most lesions are of B lineage origin with just 5%–10% being NK/T cell or Hodgkin lymphoma. The clinical presentations of PTLD following HSCT are varied, ranging from an asymptomatic rise in EBV titre on routine surveillance, to non-specific symptoms of fever and malaise, through nodal disease to fulminant and life-threatening PTLD with diffuse bulky involvement, fever, hypotension and organ dysfunction. Rarely, a distinct category of primary central nervous system PTLD is seen.

Described by Nalesnik [60], but more recently updated in the WHO Classification of tumours of haematopoietic and lymphoid tissues [56], four distinct histopathological entities are recognised. Firstly a plasmacytic hyperplasia or infectious mononucleosis-like, or “early lesion”, PTLD. Secondly, polymorphic PTLD which shows destruction of normal tissue architecture but retains a broadly polymorphic cell population. The occurrence of cytogenetic changes is limited, being seen in only 15% of cases [61]. Thirdly, monomorphic PTLD is composed of malignant cells of clonal origin. In this group of tumours, cytogenetic abnormalities are seen in up to 70% of cases, including trisomy 9, trisomy 11 and rearrangement of 8q24.1 (*MYC*) [61]. Histological classification is complex. In children, the predominant morphology is of diffuse large B cell lymphoma, although Burkitt/Burkitt-like lymphoma, Hodgkin lymphoma and plasmacytoid neoplasms are all recognised. Indeed, Hodgkin lymphoma/Hodgkin lymphoma-like PTLD is recognised as the fourth WHO category.

Following allogeneic HSCT, the risk of PTLD is relatively low, being seen in 1%–2% of recipients [27,62]. However, in patients receiving T cell depleted transplants the rate of PTLD may be as high as 24% [62], although more recent and larger series would suggest a more moderate risk, approximately 2% but still with a 3.1–15.8 fold increased risk compared to non-T cell depleted transplants [47]. The pattern of occurrence is somewhat different following HSCT as compared with solid organ transplant recipients, with onset occurring predominantly within the first year post-transplant, with a median time to onset of two months [27,63].

Management of PTLD recognises that it is primarily a deficiency of immune surveillance. The cornerstone of treatment is therefore reduction of immune suppression (RIS) and this may be sufficient to control PTLD, especially in the absence of monomorphic disease [64,65]. In the early onset situation post-HSCT, scope for RIS may be limited. Increasingly, therefore, a degree of RIS is accompanied by administration of the anti-CD20 monoclonal antibody Rituximab, either in the setting of established PTLD [66,67], or pre-emptively in response to rising EBV titres [68,69]. This particular approach is well suited to post-HSCT PTLD where the relatively short period of highest risk makes intensive monitoring of EBV viral titres feasible. Indications for more intensive treatment, principally cytotoxic chemotherapy, are poorly defined with a lack of prospective treatment trials, especially those enrolling patients post-HSCT. Indeed, those trials that do exist frequently exclude post-HSCT patients. Patients are more likely to be considered for chemotherapy if they have bulky disease, a monomorphic phenotype or failure to respond to RIS or rituximab monotherapy. Outcome amongst this group of patients is, however, poor [27,42,63,70,71]. Finally, the use of EBV-specific cytotoxic T lymphocytes (CTLs) is increasingly well investigated in small series [72], with the prospect of data from randomised trials in the near future. This approach still requires a reduction in immune suppression in order to establish effective CTL function and, at present, suffers from the time taken to generate EBV-specific cells and the cost of doing so.

## 4. Risk Factors

Several patient, disease and treatment-specific factors may contribute to the risk of a secondary malignancy in HSCT survivors. In general terms, patient risk factors include health-related lifestyle and behaviour attributes, disease factors include the underlying diagnosis that led to HSCT (e.g., FA as a cancer predisposition syndrome) and treatment risk factors include both prior (*i.e.*, months or years before HSCT) and conditioning regimen chemotherapy and radiotherapy. Many post-HSCT SMNs have multifactorial origins, for example the occurrence of PTLD in EBV-positive patients who are heavily immunocompromised either by their underlying disease (especially the severe primary immunodeficiencies [PIDs]), conditioning treatment or continued post-HSCT immunosuppressive treatment for GvHD, or in many cases a combination of these. Therefore, most studies have employed multivariate statistical analyses to account for the multiplicity of potential risk factors for the development of post-HSCT SMNs.

Although a wide variety of SMNs are reported in many different studies, solid tumours typically occur in survivors who received TBI during conditioning treatment, or other (e.g., cranial) radiotherapy during earlier treatment of their malignancy, whilst haematological SMNs, especially t-AML/MDS, are usually seen in survivors of autologous HSCT and have been attributed to extensive prior chemotherapy. PTLD usually occurs in recipients of allogeneic HSCT performed for PIDs, particularly when using mismatched donors.

### 4.1. Solid Malignancies

The impact of age as a risk factor for the development of post-HSCT SMNs has varied according to the study population. In large registry studies of childhood HSCT, younger age (<10 years) at allogeneic transplant was associated with greater risk of SMNs, especially brain and thyroid tumours (RR 3.7) [35], but age had no effect on the risk after autologous HSCT [30]. Two registry studies including both childhood and adult transplants demonstrated higher relative risks in older recipients compared to children (RR 2.0) [32,42], but a single centre study with a case control design failed to show any effect of age as a risk factor [26]. In contrast Rizzo’s very large multicentre registry study found that two thirds of brain, thyroid, bone and soft tissue SMNs occurred in patients aged <17 years at HSCT, and that age <10 years at HSCT increased the risk of solid SMN in patients receiving TBI [33]. Furthermore, age <10 years has also been shown to be a risk factor for thyroid SMNs, with a very high RR of 24.6 compared to age >20 years [40].

Several studies of the effect of conditioning have demonstrated higher risks of SMNs in patients who received TBI [27,35], or TBI or thoraco-abdominal radiotherapy [40]. As mentioned above, the risk was amplified considerably in younger recipients in Rizzo’s study, with a RR of over 50 in those receiving TBI at <10 years [33]. In contrast, no increased risk due to TBI was observed after paediatric autologous HSCTs [30], nor in a large combined paediatric/adult single centre study of both autologous and allogeneic HSCTs, although the fact that 78% of patients had received TBI or total lymphoid irradiation may have limited this study’s power [42]. The single centre cohort/case control study mentioned above failed to show any overall effect of TBI on the risk of SMNs, but did find that most thyroid, liver and oral cancers occurred in TBI recipients [26]. Previous cranial radiotherapy (*i.e.*, given during earlier antileukaemic treatment) may contribute to the later development of brain and thyroid SMNs [35].

Few studies have specifically investigated the effect of patient gender on post-transplant SMNs. Females have a higher risk of thyroid SMNs [26,40], perhaps unsurprisingly given the higher prevalence of thyroid cancer in females in the general population. In contrast, higher risks of skin and oral/pharyngeal squamous cell carcinomas (SCCs) have been described in males [26,33]. Danner-Koptik [30] found no influence of gender on the risk after paediatric autologous HSCT.

The influences of cGvHD and its treatment, particularly on mucocutaneous SMNs, have also been investigated with varied findings. Socie’s registry study of paediatric HSCT [35] found that cGvHD reduced the risk of solid SMNs (RR 0.2) but two large combined paediatric/adult single centre studies found no effect [26,27]. In contrast, two very large multicenter international registry studies have shown that cGvHD increased the risk of thyroid (RR 2.9), skin (RR 11.0) and oral (RR 5.3) SMNs [33,40]. Furthermore, GvHD is clearly the main risk factor for the development of solid SMNs post-HSCT in patients with FA [17]. Immunosuppressive treatment of cGvHD with ciclosporin (RR 2.5) or thalidomide (RR 3.4) has been reported to be an independent risk for developing SMNs in a large combined (all ages and all HSCT types) registry study [32]. Overall, cGvHD appears to have an important role as a risk for mucocutaneous SMNs post-HSCT.

Unsurprisingly, given its known role in the aetiology of hepatocellular carcinoma in the general population, chronic hepatitis C infection has been reported as an additional risk factor for the development of post-HSCT liver cancer [26].

### 4.2. Myeloid Malignancies

Therapy-related AML/MDS is largely, although not exclusively [42], a second neoplasm associated with autologous transplantation. This underpins the generally accepted view that the principle mechanism of malignant transformation is the genotoxic stress of DNA damaging agents used either for prior therapy, stem cell mobilisation or conditioning (which is frequently not myeloablative). Pre-existing DNA damage in engrafting stem cells is compounded by a high replicative stress, either prior to or following transplantation. In keeping with this, a number of risk factors have been identified which relate to exposure to specific cytotoxic chemotherapy agents or replicative burden. However, quantifying the relative effects of pre- and peri-transplantation risk factors remains difficult.

The best defined risk factors for t-AML/MDS are the use of topoisomerase II inhibitors, alkylating agents and radiotherapy. Amongst these, etoposide and cyclophosphamide are the most commonly used chemotherapeutic agents in paediatric practice. As described above, each has a characteristic presentation and associated cytogenetic abnormalities. Indeed, use of both drugs is associated with therapy-related myeloid malignancy and the use of HSCT [55,73]. Beyond the absolute risk associated with the use of, particularly, epipodophyllotoxins, there is ongoing debate regarding the relative contribution of total dose, with a threshold of 2000 mg/m^2^ etoposide often cited [74], *versus* schedule of treatment [75]. This has been especially hard to define further, as different doses and schedules are commonly compared across disparate trials and in combination with different additional cytotoxic drugs. However, one recent report of 87 children undergoing treatment for high risk neuroblastoma demonstrated a high incidence of t-AML/MDS, with a 15 year cumulative incidence of 34.2% [45]. The authors attribute this to the very intensive use of etoposide and cyclophosphamide in both of the regimens studied (CP1—Cyclophosphamide 10 g/m^2^, etoposide 6400 mg/m^2^; CP2—Cyclophosphamide 19.4 g/m^2^, etoposide 3302 mg/m^2^) combined with the late harvesting of HSCs. Finally, in children treated for relapsed acute lymphoblastic leukaemia, multivariate analysis identified the use of etoposide and doses of cyclophosphamide >3000 mg/m^2^ to be independently associated with the risk of developing t-AML/MDS [76].

The use of radiation therapy, and particularly total body irradiation (TBI) as part of conditioning regimens for autologous transplantation is relatively uncommon in children today. However, some, but not all, adult studies have found an increased risk of t-AML/MDS following TBI, especially at higher doses [77]. If true, these data provide an interesting perspective, as the effect of TBI must be mediated either by residual HSCs surviving conditioning or by changes in the microenvironment. However, a recent study found no association between TBI and the risk of t-AML/MDS in children [30].

Several studies have identified initial diagnosis of both Hodgkin lymphoma (HL) and non-Hodgkin lymphoma (NHL) as risk factors for developing a therapy-related myeloid malignancy. This is in accordance with the importance of prior treatment, as lymphoma patients have frequently received significant doses of prior chemotherapy and many will undergo autologous transplantation following relapse and second line chemotherapy. An Australian BMT registry-based study looked at 7,765 autologous transplants in patients over 14 years of age and identified a within cohort SIR of t-AML/MDS of 26.4 for patients treated for NHL and 35.4 for HL [28]. The combined paediatric/adult single centre study by Bhatia identified an increased cumulative probability of t-AML/MDS in lymphoma patients, being 13.5% at six years post-transplant, compared to an actuarial risk of t-AML/MDS across the whole group of 2.1% at nine years [27]. However, this finding was not identified in the subsequent updated study [42] and has not been reproduced in children. Indeed, a large multicentre study of children undergoing autologous HSCT did not identify an increased risk in those treated for lymphoma [30]. However, in children the indications for autologous transplant differ from those in adults and it is noteworthy that each of the three principle indications for autologous transplant in Danner-Koptik’s study, namely lymphoma, sarcoma and neuroblastoma, have pre-transplant induction chemotherapy backbones containing significant doses of both topoisomerase II inhibitors and alkylating agents.

The RR associated with each stem cell source is an interesting characteristic which is not fully understood. A number of reports have shown an increased risk of t-AML/MDS following autologous peripheral blood stem cell (PBSC) transplantation as compared with autologous marrow transplantation. Bhatia identified that, within the group of patients receiving autologous transplant for lymphoma diagnoses, those receiving PBSC had a significantly higher incidence of t-AML/MDS than those receiving autologous marrow (35.8% *versus* 4.1% at four years, *p* = 0.004) [27]. This was confirmed by Baker in their follow-up report from the same institution [42] and in a separate cohort of 467 Hodgkin lymphoma patients (RR 3.1, *p* = 0.03) [78]. However, not all adult data support this finding [28] and the same effect has not been shown in paediatric studies, although this may purely reflect the much smaller number of children receiving autologous transplants and the predominant use of PBSC in modern high dose regimens. Possible explanations for this finding are that stem cells mobilised into the peripheral circulation following chemotherapy are likely not to have completed DNA repair and therefore may carry mutations forward into a situation with a high replicative drive, both endogenous and exogenous, as growth factors such as G-CSF are frequently used after autologous HSCT. In contrast, marrow stem cells are commonly harvested in steady state and therefore less likely to harbour mutations. Alternatively, it may be that mobilisation preferentially affects mutated/pre-leukaemic stem cells, resulting in a greater proportion of these cells in the harvested product. Either of these explanations may hold an equal relevance to paediatric patients and merit further investigation.

Many of the risk factors discussed above have clear associations with the presumed mechanism of stem cell mutation and replicative stress, and the hallmark clinical and genetic features of both topoisomerase II inhibitor and/or alkylating agent induced t-AML/MDS seen in children. However, it is noteworthy that little evidence exists to demonstrate the role of modifiable risk factors of HSCT itself, as opposed to prior chemotherapy, specifically in the paediatric population. The intensive pre-transplant treatment of neuroblastoma provides a good example of the importance of differentiating prior treatment from transplantation specific factors [45]. Furthermore, the largest multicentre paediatric cohort to report autologous transplantation outcomes failed to identify any additional risk attributable to a number of established or postulated adult risk factors, including age, gender, diagnosis, use of either TBI or etoposide as conditioning or number of autologous transplants [30].

### 4.3. Post-Transplantation Lymphoproliferative Disorder

The pathogenesis of PTLD is believed to centre on the lack of T cell-mediated suppression of EBV-infected B lymphocytes. In the absence of the immune surveillance offered by EBV-specific T cells, latently infected B cells (most frequently of donor origin) are able to proliferate unchecked and are, therefore, more likely to acquire the additional genetic mutations promoting development of polymorphic or monomorphic proliferations. The risk factors for post-HSCT PTLD relate therefore to the degree of suppression of EBV-specific T cell numbers and function in comparison with the EBV-positive B-cell load.

In contrast to an overall incidence of PTLD following HSCT of approximately 1%, several studies have shown that donor T cell-specific lymphocyte depletion, both *in vitro* pre- and *in vivo* post-transplant, increases this risk to as much as 24% [62,79]. In a large single centre study of adults and children in the US, Bhatia identified a cumulative probability of developing PTLD by 10 years of 11.4% following T cell depletion for GvHD prophylaxis and 11.3% following the use of ATG during conditioning [27]. The subsequent update of this center’s experience similarly identified a RR of 4 following T cell depletion and 3.7 following the use of ATG either as part of conditioning or treatment for acute GvHD (aGvHD) [42]. Similar RRs were identified in the multicentre paediatric report from the IBMTR for both T cell depletion (RR 4.8) and use of ATG (RR 3.1) [35]. A clear difference exists between the greater scale of risk of those methods which selectively deplete T cells or T and NK cells only (RR 8.4–15.8) when compared with approaches which unselectively remove both B and T lymphocytes, such as Alemtuzumab (RR 3.1–3.2) [47].

A factor related to both conditioning approach and graft manipulation is that of donor matching. Both single institution and large multicentre registry-based studies have demonstrated a greater RR for developing PTLD following unrelated donor and mismatched donor transplants. Bhatia and this group’s updated report presented by Baker identified a RR of 8.9 for mismatched transplants and 9.0 for mismatched related transplants respectively [27,42]. Socie also reported a raised risk from unrelated and mis-matched related donors, with a RR of 7.5 [35]. However, the complex interactions of donor choice, graft manipulation and GvHD prophylaxis/treatment mean caution is required in interpreting the independence and importance of these factors. Amongst nearly 27,000 HSCT recipients, an interaction existed between donor selection (unrelated or mismatched related) and T cell depletion or ATG use [47]. In this study, recipients of unrelated or greater than 2 HLA-mismatched grafts who did not receive T cell depletion or ATG had a RR of PTLD of 0.9. However, the use of an unrelated or mismatched (>2 loci) donor significantly enhanced the pre-existing risk from T cell depletion, ATG or both by 3.8 fold.

The impact of GvHD on the risk of developing PTLD is equally complex to define. The larger studies already examined have given quite varied estimates of the risk associated with GvHD. In part, this may relate to the complex interplay of donor selection, graft manipulation and conditioning, discussed in the previous paragraph. These factors are compounded by the subsequent management of GvHD which will impose a further burden of immunosuppression on the patient. Whilst Bhatia showed a substantial risk from grade IV aGvHD (RR 9.4) [27], the more recent update of this cohort identified a rather more modest effect (RR 2.4) with grade III or IV aGvHD [42]. Neither study identified any effect from cGvHD. However, Socie failed to identify any risk from aGvHD in 3,182 children receiving allogeneic HSCT for acute leukaemia, but did identify a RR of 6.5 with moderate or severe cGvHD [35]. One study has suggested that cGvHD may be associated with later onset PTLD [46], although this may represent a different disease entity to the early onset PTLD most commonly seen after HSCT. An update of this large, multi-institutional study identified a small but significant impact of both acute and chronic GvHD (RR 1.7 and 2, respectively) [47].

In keeping with the hypothesis that PTLD is driven by the lack of EBV-specific T cell immunity in the face of EBV-immortalised B cell persistence, the increasing use of non-myeloablative or reduced intensity conditioning (RIC) regimens appears to be associated with an increased risk of PTLD [80]. Not only does it increase the risk of recipient B cells surviving conditioning, but tolerance of the graft is maintained by use of more intensive immunosuppression post-transplant. This results in prolonged time to recovery of EBV-specific T cell immunity. In this setting, the use of ATG in the conditioning regimen identified a group with an incidence of PTLD of 21%, significantly higher than those receiving either myeloablative conditioning (3%) or non-myeloablative conditioning without ATG (2%) [81]. These risks are compounded by their use in patients with primary immune deficiency who, as mentioned above, have a substantially higher risk of second malignancy following HSCT. Furthermore, whilst not identified as a risk factor following myeloablative conditioning, the use of cord blood as a stem cell source increases the risk of PTLD following RIC transplants [81].

Whilst a complex interplay clearly exists between multiple factors involved in immunosuppression post HSCT, all these factors support the central hypothesis that PTLD is the result of poor EBV-specific T cell mediated immune reconstitution. Furthermore, the presence of multiple risk factors results in a multiplicative effect on an individual’s risk [46,47]. For a specific patient, this allows some personalisation of risk of developing PTLD in the setting of, for example, asymptomatic EBV viraemia. Further studies may allow for more focused use of pre-emptive immunotherapy (e.g., Rituximab) based on the presence or absence of each of the risk factors.

### 4.4. Fanconi Anaemia

FA is associated with an increased risk of primary MDS or AML, usually occurring during the first three decades of life, whilst solid tumours are reported increasingly as primary or second malignancies during longer follow-up, often but not exclusively in survivors of HSCT. SCCs of the head, neck and anogenital regions are the commonest solid tumours reported, but hepatic, brain, renal, skin and breast malignancies are also described [16,82,83,84]. A North American retrospective cohort of 145 FA patients described a cumulative incidence of 39% for malignancy (10% leukaemia, 29% solid tumour) by 48 years of age. Forty-four FA patients had undergone HSCT and the crude rate for solid tumours prior to HSCT, AML, or death was 0.7% per year, and 1.99% after HSCT [16]. The rate ratio was 2.79 (*p* = 0.07), but the authors commented that the small number of events and person-years in the HSCT group limited the utility of this comparison. A German FA Registry study, comprising 181 FA patients, reported very similar findings, with a 28% incidence of solid tumours by 49 years of age, a higher risk of AML (22% at 36 years age), and a non-significant (*p* = 0.11) trend to an increased risk (hazard ratio 3.8) of solid tumours after HSCT [84]. Although radiotherapy (during HSCT conditioning) had previously been regarded as a major risk factor for secondary solid tumours in FA, more recent evidence has not demonstrated this [44] and indeed there is clear evidence that the occurrence of acute or chronic GvHD is the most important factor [17,85].

### 4.5. Other Cancer Predisposition Syndromes

Several other extremely rare constitutional bone marrow failure syndromes which are indications for HSCT are also regarded as cancer predisposition syndromes. Malignancy, usually solid tumours in the third and fourth decades of life, occurs in 10%–15% of dyskeratosis congenita patients, whilst the risk of myeloid malignancy (AML/MDS) is increased in Schwachman-Diamond syndrome, congenital amegakaryocytic thrombocytopenia and severe congenital neutropenia (Kostmann syndrome). Both solid tumours and myeloid malignancy occur in patients with Diamond-Blackfan anaemia. Due to their rarity, there is no information about the influence of HSCT on these risks [86].

## 5. Future Research Questions

With SMNs already known to be a major cause of morbidity and mortality post-HSCT, and with the true ongoing longer-term risk of solid SMN as yet not quantified, further studies are required to provide a strong evidence base for risk reduction, surveillance and management of SMNs. This must include determining more clearly the modifiable risk factors associated with SMNs. Clinical trials will then be required to study the impact of reduced exposure to these factors by, for example, the increased application of non-TBI and non-myeloablative conditioning regimens, reduced use of epipodophyllotoxins and/or alkylating agents by improved patient stratification, or improved donor matching and hence reduction of GvHD. Of course, limiting exposure to the principle risk factor, the need for HSCT, would provide the greatest modification in risk. This may be achievable through better patient stratification and delivery of appropriate initial treatment intensity, application of molecularly targeted therapies to reduce exposure to DNA damaging agents or even replacement of allogeneic donor with alternative, patient-derived stem cell technologies. It must be recognised, however, that for many patient groups such as high risk AML or neuroblastoma, the greatest threat to successful treatment remains relapse. Efforts to modify the risk of developing an SMN must not significantly affect the chance of primary disease control.

Some risk factors remain, despite our best efforts, but being able to stratify patients based on their risk of developing an SMN post-HSCT will allow the focusing of surveillance resources and also reduce anxiety for those identified to be at lower risk. As survival increases and with no plateau for the risk of solid SMN yet identified, lifelong surveillance is required and so appropriate use of resources will become increasingly important. Existing long-term follow-up guidelines differ considerably in the nature and frequency of surveillance investigations recommended for survivors of childhood malignancy and HSCT, leading to uncertainty about optimum screening practice [11]. Efforts to achieve greater consistency and strength of recommendations have been exemplified recently by the provision of an evidence base for the required intensity and optimum modality for surveillance for secondary breast cancer, permitting a consistent approach across units [49].

Finally, many SMNs still have a poorer prognosis than their sporadic counterparts [27,35,42]. This may result from the prior treatment of patients and resultant existing toxicities, but equally may reflect the common occurrence of high risk disease, characterized by recurrent high risk cytogenetic features, in this group of patients. Either way, improved risk stratification of SMNs combined with an increasing interest in their identification and management is required to improve the survival in these patients. One important area for further investigation is the development of a risk-adapted approach to pre-emptive treatment of EBV-driven PTLD. Using patient risk features and clinical characteristics, better definition can be given to the need for intervention with Rituximab or a similar agent, *versus* a sustained monitoring approach.

In the longer term, improved understanding of the molecular pathogenesis of post-HSCT SMNs may enable safer use of the causative cytotoxic agents and ultimately improved treatment of the SMNs.
